# Kinetics of plasmatic cytokines and cystatin C during and after hemodialysis in septic shock-related acute renal failure

**DOI:** 10.1186/cc9064

**Published:** 2010-06-14

**Authors:** Nicolas Mayeur, Lionel Rostaing, Marie B Nogier, Acil Jaafar, Olivier Cointault, Nassim Kamar, Jean M Conil, Olivier Fourcade, Laurence Lavayssiere

**Affiliations:** 1 Anesthesia and Intensive Care Unit Department, GRCB 48, Purpan University Hospital, Place du Dr Baylac, TSA 40031, 31059 Toulouse Cedex 9, France; 2 Department of Nephrology, Dialysis and Transplantation, Intensive Care Unit, Rangueil University Hospital, 1 Avenue Jean Poulhès, TSA 50032, 31059 Toulouse Cedex 9, France; 3 Department of Clinical Physiology, Rangueil University Hospital, 1 Avenue Jean Poulhès, TSA 50032, 31059 Toulouse Cedex 9, France

## Abstract

**Introduction:**

Cystatin C could be a relevant residual glomerular filtration rate marker during hemodialysis (HD), and a high cytokine plasma (p) rate is associated with an increase in mortality during sepsis. To the best of our knowledge, cytokines and cystatin C kinetics during and after HD during sepsis have never been studied. In this study, we described p cytokines and cystatin C variations during and after hemodialysis in septic-shock patients with acute kidney injury (AKI).

**Methods:**

Ten patients, from two tertiary ICUs, with septic shock-related AKI, according to RIFLE class F, were studied. In this prospective observational study, blood samples were collected at the start, after 1 hour, 2 hours, and at the end of HD with a polymethymethacrylate (PMMA) hemodialyzer (D0, D1, D2, and endD), and 30, 60, 90, 120, and 180 min after HD (postD0.5, postD1, postD1.5, postD2, and postD3). We measured p interleukins (IL)-6, IL-8, IL-10, cystatin C, and albumin. Results are expressed as variations from D0 (mean ± SD).

**Results:**

During HD, p[IL-6] did not vary significantly, whereas p[IL-8] and p[IL-10] reductions by D1 were 31.8 ± 21.2% and 36.3 ± 26%, respectively (*P *< 0.05 as compared with D0). At postD3, p[IL-8] and p[IL-10] returned to their initial values. p[Cystatin C] was significantly reduced from D1 to postD1, with a maximal reduction of 30 ± 6.7% on D2 (*P *< 0.05). Norepinephrine infusion rate decreased from D0 to postD3 (0.65 ± 0.39 to 0.49 ± 0.37 μg/kg/min; *P *< 0.05).

**Conclusions:**

HD allows a transient and selective decrease in p cytokines, which are known as being correlated with mortality during septic shock. Because of a significant decrease in p cystatin C during HD, this should not be considered as an accurate marker for residual glomerular filtration rate during septic acute renal failure when receiving HD with a PMMA hemodialyzer.

## Introduction

Sepsis is the leading cause of acute kidney injury (AKI) [1]. The combination of sepsis and acute renal failure is associated with high mortality and morbidity [1]. AKI treatment mostly requires renal-replacement therapy (RRT). Two modalities of RRT are available in intensive-care units: continuous RRT (CRRT) using venovenous hemodiafiltration/hemofiltration or intermittent RRT (IRRT) using hemodialysis.

Sepsis causes systemic inflammatory response syndrome (SIRS), mediated by many biologically active inflammatory mediators (including cytokines like interleukins) [2,3]. High plasma interleukin (IL) levels are associated with increased mortality in human septic AKI (that is, IL-6, IL-8, and IL-10) [4-8] and might contribute to the pathogenesis of sepsis-related organ failure, including AKI [2,9]. Unfortunately, therapies targeting particular components of the SIRS-associated cytokine network have failed, probably because of the dynamic complexity of SIRS [10-12]. New data suggested that RRT could modulate SIRS via nonspecific extracorporeal removal of cytokines: this has renewed interest in this mediator-directed therapy [13,14].

In contrary to CRRT, sparse information is available concerning IRRT and cytokine levels during sepsis. Haase *et al*. [15], in a preliminary study, suggested that the use of hemodialyzers with high-molecular-weight cutoff membranes may lead to significant removal of plasma cytokines during hemodialysis. However, to the best of our knowledge, data concerning the kinetics of plasma cytokines after hemodialysis in sepsis patients with AKI are dramatically lacking. In patients with chronic renal failure, IRRT is followed by a fast but mild increase in serum urea or potassium levels during the first hour ("rebound" phenomenon) [16,17]. Similarly, given the high level of cytokine production in septic tissues, plasma cytokine levels may dramatically vary after IRRT.

Estimation of residual glomerular filtration rate (rGFR) in patients with AKI is also a major concern in the ICU. Cystatin C is a better marker of GFR than is serum creatinine in chronic kidney disease, but its involvement in ARF is still controversial [18,19]. Cystatin C is a middle-mass molecule (≈13 kDa) that is not supposed to be removed by the standard hemodialyzer. This characteristic may be of interest when evaluating rGFR, and several studies suggested that cystatin C could be used as an rGFR marker during peritoneal dialysis and intermittent hemodialysis [20,21]. As cytokines, plasma variations of cystatin C during IRRT for septic shock-related acute renal failure have not been studied. In this prospective observational study, we assessed the per- and postdialysis kinetic plasma levels of IL-6, IL-8, IL-10, cystatin C, and albumin in ten patients with septic shock-related AKI that required RRT.

## Materials and methods

### Setting and eligibility

This study was a prospective observational case series, conducted from September 2007 to December 2008 in the nephrologic and transplantation intensive care unit (ICU) and the polyvalent ICU units at Toulouse University Hospital (France). To be included in the study, patients had to reach the following criteria: (1) severe sepsis or septic shock of <24 h, as defined by the criteria of the American College of Chest Physicians/Society of Critical Care Medicine Consensus Conference [22]; (2) a need for renal-replacement therapy defined as Failure according to the RIFLE criteria [23] (oliguria < 0.3 ml/kg/h during 24 hours, or anuria during 12 hours, or threefold increase in creatinemia); and (3) an age of older than 18 years. Exclusion criteria were as follows: pregnancy, previous chronic renal failure requiring hemodialysis, liver cirrhosis, acute pancreatitis, organ transplantation, and/or immunosuppressive therapy. As requested by our local institutional research committee (Centre Hospitalier Universitaire de Toulouse, Toulouse, France), after approval, informed consent was obtained from each patient's next of kin. This study was performed in accordance with the Helsinki declaration.

The following data for all patients were recorded: age, gender, diagnosis, SAPS 2, and SOFA scores at inclusion. Biomarkers and treatments received were collected from the start of HD, at the end of HD, and for 3 hours after the end of HD. Arterial line and central venous catheters allowed documentation of mean arterial pressure (MAP) and drug infusions, respectively. Cardiac-output measurements were obtained, if necessary, via transthoracic echography or a Pulse-Indexed Continuous Cardiac Output (PiCCo) monitor (Pulsion; Medical Systems AG, Munich, Germany). Throughout the ICU hospitalization, the patients were resuscitated, if needed, to reach hemodynamic goals as recommended by the international guidelines for septic shock and were under the responsibility of an ICU-qualified senior physician [24]. Blood cultures and specific bacterial samples were collected at various times to specify the etiology of the infection. If known, the site of infection was recorded. None of the patients received enteral or parenteral nutrition before, within, and for the 3 hours after dialysis. Supplements of trace elements, water, and fat-soluble vitamins were given. Continuous intravenous insulin therapy was delivered if necessary to achieve a normal glycemia (range, 1 to 1.5 g/L).

### Protocol and intermittent renal-replacement therapy

D0 was considered to be the start of hemodialysis. Mean arterial pressure (MAP), heart rate (HR), dobutamine and/or norepinephrine dose, and fluid infusion were recorded at D0, at every hour during hemodialysis (D1; D2), at the end of HD (endD), and at 30, 60, 90, 120, and 180 minutes after completing HD (postD0.5, postD1, postD1.5, postD2, and postD3, respectively). During hemodialysis, conductivity, K_t_, and dialysance were recorded.

According to the literature, hemodynamic stability during hemodialysis has been optimized by using several methods: arterial- and venous-circuit simultaneous connection, high conductivity, an ultrafiltration-free first hour, circuit-to-body temperature difference of 2°C, and high dialysate calcium concentration (1.75 m*M*) [25].

Blood flow was started at 250 ml/min and was enhanced according to hemodynamic tolerance. Ultrafiltration was based on the individual patient's fluid status. Duration of HD was 3 hours.

Vascular access for renal replacement was obtained by using a double-lumen venous cannula (Hemoaccess 13 F, 25 cm, Hospal). An Integra generator was used for the hemodialysis (Hospal; Gambro Renal Products, Antwerp, Belgium). We used a polymethylmetacrylate (PMMA) membrane: Filtrizer BK-1, 6F; Toray Industries, Tokyo, Japan. The ability of the PMMA membrane to remove cytokines during both IRRT for chronic renal failure and CRRT during septic AKI has been described [26,27]. The PMMA dialyzer has been reported to adsorb proinflammatory cytokines or free light chains during multiple myeloma and is specific insofar as it can remove proteins by adsorption as well as permeation [28-30]. The extracorporeal circuit was anticoagulated with a continuous unfractionated heparin infusion, with the anticoagulation regimen adjusted to the individual patient's needs.

### Biologic and cytokine analyses

Blood samples were collected in nonheparinized tubes and were immediately centrifuged in the ICU at 4,000 rpm for 10 minutes (4°C). Plasma was subsequently stored at -70°C until assayed. Cystatin C measurements were obtained by using PETIA (particle-enhanced turbidimetric immunoassay) with a cystatin C reagent (Cys C Immunoparticles; Dako Inc., Glostrup, Denmark) on a ABXPentra 400 chemistry analyzer (Horiba Medical, Kyoto, Japan). Serum albumin concentrations were quantified by nephelometry (Immage 800; Beckman Coulter, Villepinte, France). Plasma (p) cytokine levels were measured with enzyme-linked immunosorbent assays (ELISAs), according to the manufacturer's instructions (BD-Biosciences, Le Pont De Claix, France).

### Measurements and statistics

Continuous variables during and after dialysis were compared by using Friedman nonparametric tests. If significant, the Dunn *post hoc *test was applied. Univariate analysis of D0 data from survivors and nonsurvivors was performed by using the nonparametric Mann-Whitney test. Cytokines, albuminemia, and cystatin C were expressed as relative concentration from baseline value (D0). Results are expressed as mean ± standard deviation. A *P *value < 0.05 was considered statistically significant. The data were analyzed by using GraphPad Prism (version 4 2005; Graphpad Software Inc., San Diego, CA, USA).

## Results

### Patients and hemodialysis

Ten patients with septic shock in whom AKI developed were enrolled in this study. Their main demographic and clinical data at baseline are summarized in Tables 1 and 2.

**Table 1:** Characteristics of patients

Characteristics	Data
Age (years)	67.40 ± 7.21
Sex ratio (F/M)	3/7
SAPS 2 at D0	79.11 ± 4.73
SOFA at D0	14.6 ± 0.8
Mechanical ventilation	9/10
Norepinephrine	10/10
Dobutamine	1/10
In-hospital mortality (%)	60
Infectious disease: localization	
Pulmonary	5/10
Peritoneal	3/10
Cutaneous	2/10
Urologic	1/10
Bilirubinemia at D0 (mg/L)	19.9 ± 19.1
Factor V at D0 (%)	64.2 ± 24.1
CRP at D0 (mg/L)	240.8 ± 103.9
Leukocytes at D0 (cells/μl)	23,981 ± 13,726
Hemoglobinemia at D0 (g/L)	10.9 ± 0.38
p[IL-6] at D0 (pg/ml)	840 ± 539
p[IL-8] at D0 (pg/ml)	724 ± 572
p[IL-l0] at D0 (pg/ml)	178 ± 173

F, female; IL, interleukin; M, male; SAPS 2, Simplified Acute Physiology Score; SOFA, Sequential Organ Failure score.

Values expressed as mean ± standard deviation.

**Table 2:** Characteristics of patients at D0, endD, and post-D3

Characteristics	D0	EndD	Post-D3
Cystatin C (mg/L)	3.73 ± 1.17	2.65 ± 0.74^a^	3.25 ± 0.94
Urea (m*M*)	28.8 ± 10.7	14.8 ± 6.3^a^	17.4 ± 7.3
Creatinemia (μ*M*)	399 ± 148	230 ± 74	257 ± 83
Bicarbonatemia (m*M*)	15.7 ± 4	22.9 ± 1.8^a^	21.7 ± 4^a^
Albuminemia (g/L)	18.8 ± 4.5	19.9 ± 5.3^a^	17.7 ± 5.1
Norepinephrine rate (μg/kg/min)	0.65 ± 0.12	0.57 ± 0.38	0.49 ± 0.37
MAP (mm Hg)	80.1 ± 13.7	80.7 ± 10.8	81 ± 16.4
Heart rate (beats/min)	110 ± 20	113 ± 19	103 ± 17
Urinary output (ml/h)	17 ± 14.9	15 ± 12	24.5 ± 17.8
Lactatemia (m*M*)	3.2 ± 1.8		2.4 ± 1.5
pH	7.27 ± 0.08		7.39 ± 0.05^b^
SvO_2 _(%)	77.2 ± 10.3		77.9 ± 12

MAP, mean arterial pressure; SvO_2_, mixed venous oxygen saturation. Results are expressed as the mean ± standard deviation. ^a^*P *< 0.05 versus D0 (Friedman test). ^b^*P *< 0.05 versus D0 (Wilcoxon signed-rank test).

**Figure 1:**
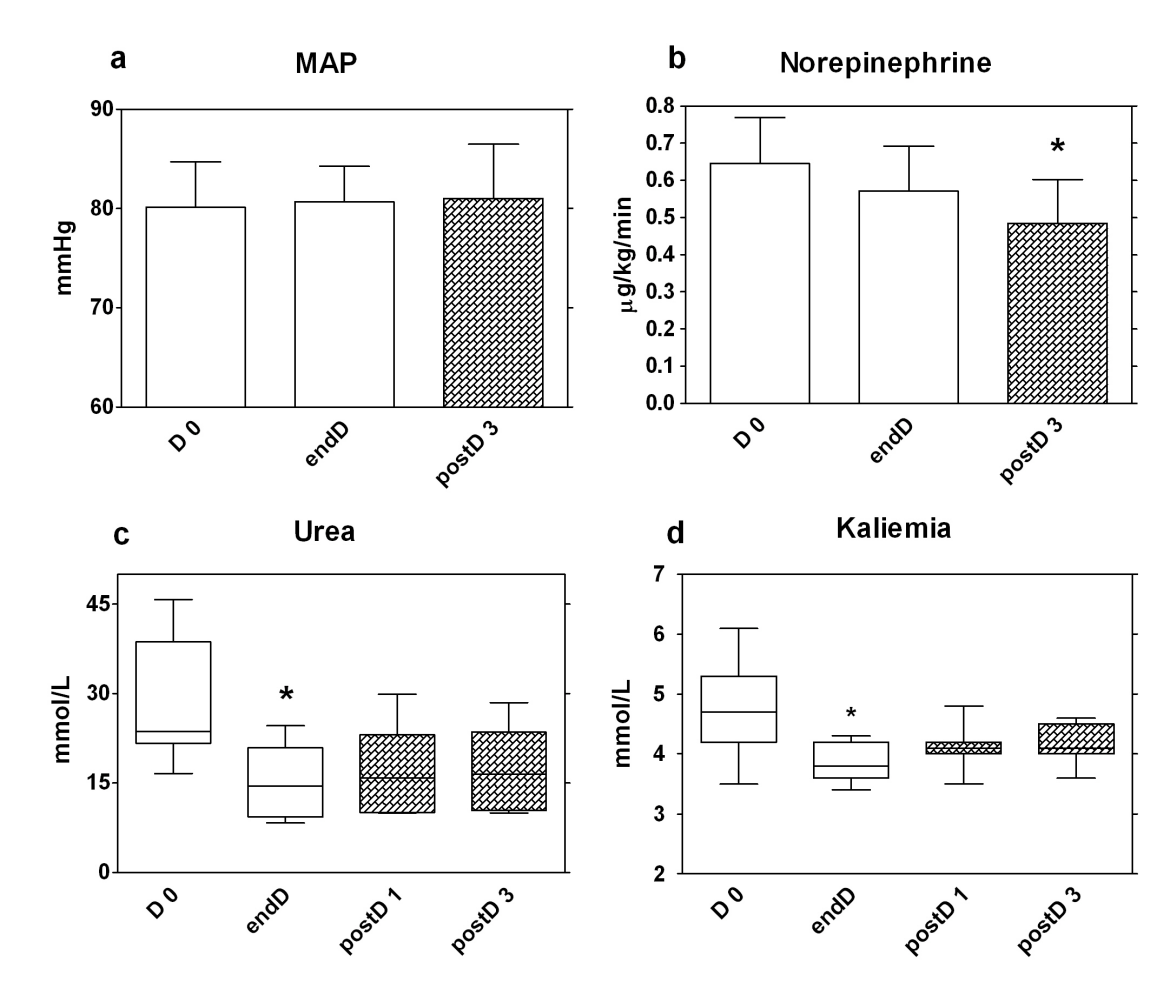
**Hemodynamic, urea, and kalemia variations during and after hemodialysis**. Mean arterial pressure **(a) **and norepinephrine infusion rate **(b) **at the start (D0), the end (endD), and 3 hours (postD3) after hemodialysis. Values are expressed as mean ± SEM. Urea **(c) **and kalemia **(d) **at the start, the end, 1 hour, and 3 hours after hemodialysis, D0, end D (clear), postD1 and postD2 (dark), respectively. Values are expressed in boxplots. *P < 0.05; Friedman test.

In brief, intermittent hemodialysis was not deleterious to their hemodynamics, as suggested by the stability of MAP (see Figure 1a). A significant decrease was observed in the norepinephrine infusion rate during the 6 hours of the study (0.65 ± 0.39 vs. 0.49 ± 0.37 μg/kg/min; *P *< 0.01; Figure 1b). Fluid loading between D0 and endD, and between endD and postD3 was 75 ± 169 and 125 ± 143 ml, respectively. This low fluid intake during IRRT is explained by the mixed venous oxygen saturation (SvO_2_) being high at D0 (77.2 ± 10.3 mm Hg) after previous adequate resuscitation. Atrial fibrillation (AF) was apparent in four patients at D0. AF resolved in one patient at postD1, but persisted in the other three despite hemodynamic improvement.

Only one patient required 1,900 ml of ultrafiltration. Conductivity at D0 was 146 ± 0.43 mEq/L. Mean dialysance and K_t _were 152 ± 17 and 28 ± 4.8, respectively. The urea-reduction fraction was 48.5% (*P *< 0.003; Figure 1c).

Last, in-hospital mortality was 60%. Also, at D0, no difference was noted between survivors and nonsurvivors according to plasma IL-6, IL-8, and IL-10 levels and other biologic characteristics (data not shown).

### Cytokines, albumin, and cystatin C kinetics

**Figure 2:**
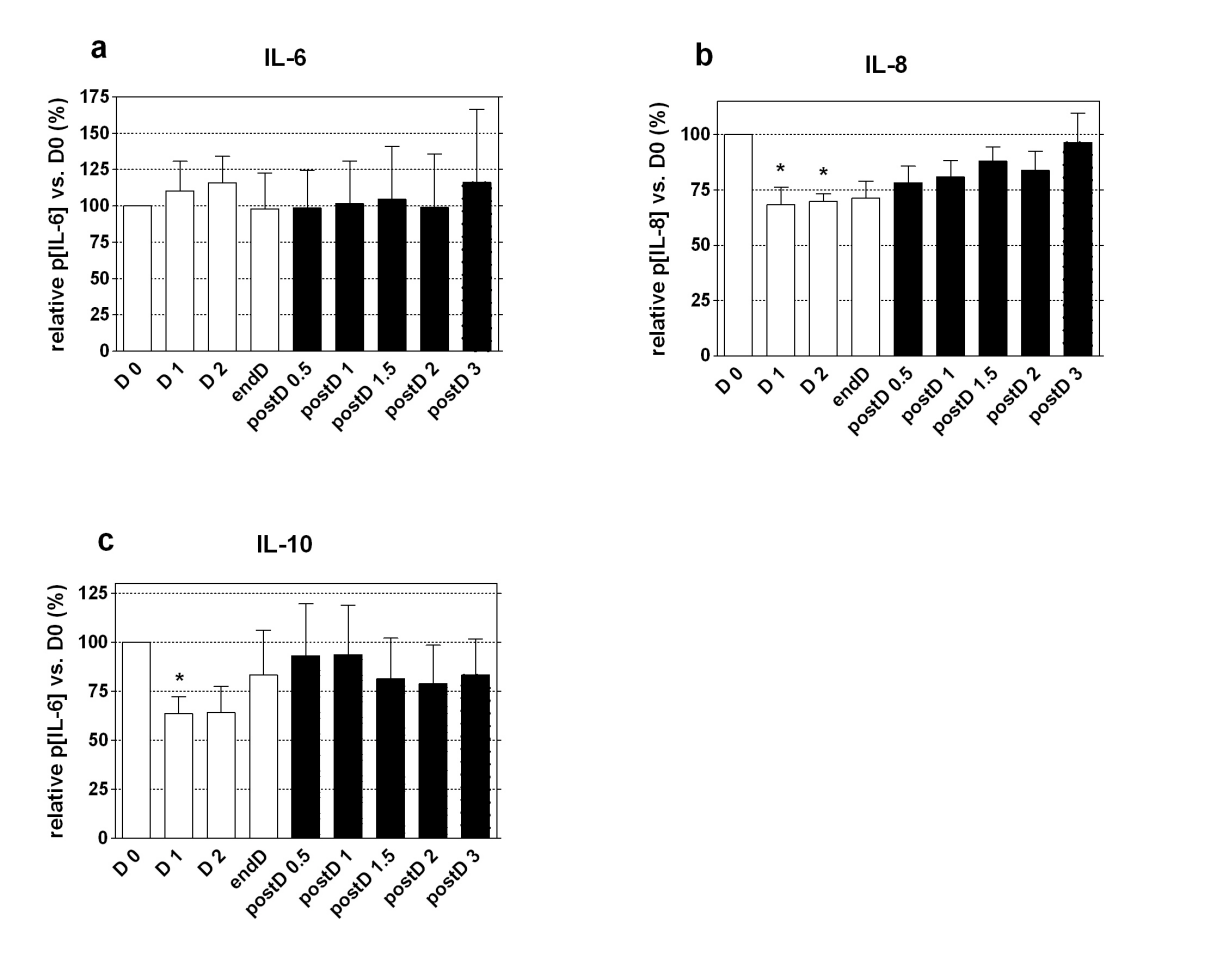
**Cytokine variations during hemodialysis**. Plasma level of IL-6 **(a)**, lL-8 **(b)**, and IL-10 **(c)**. Results are expressed in percentage of value at D0 during (clear) and after (dark) hemodialysis as mean ± SEM. *P < 0.05 versus D0. Friedman test.

**Figure 3:**
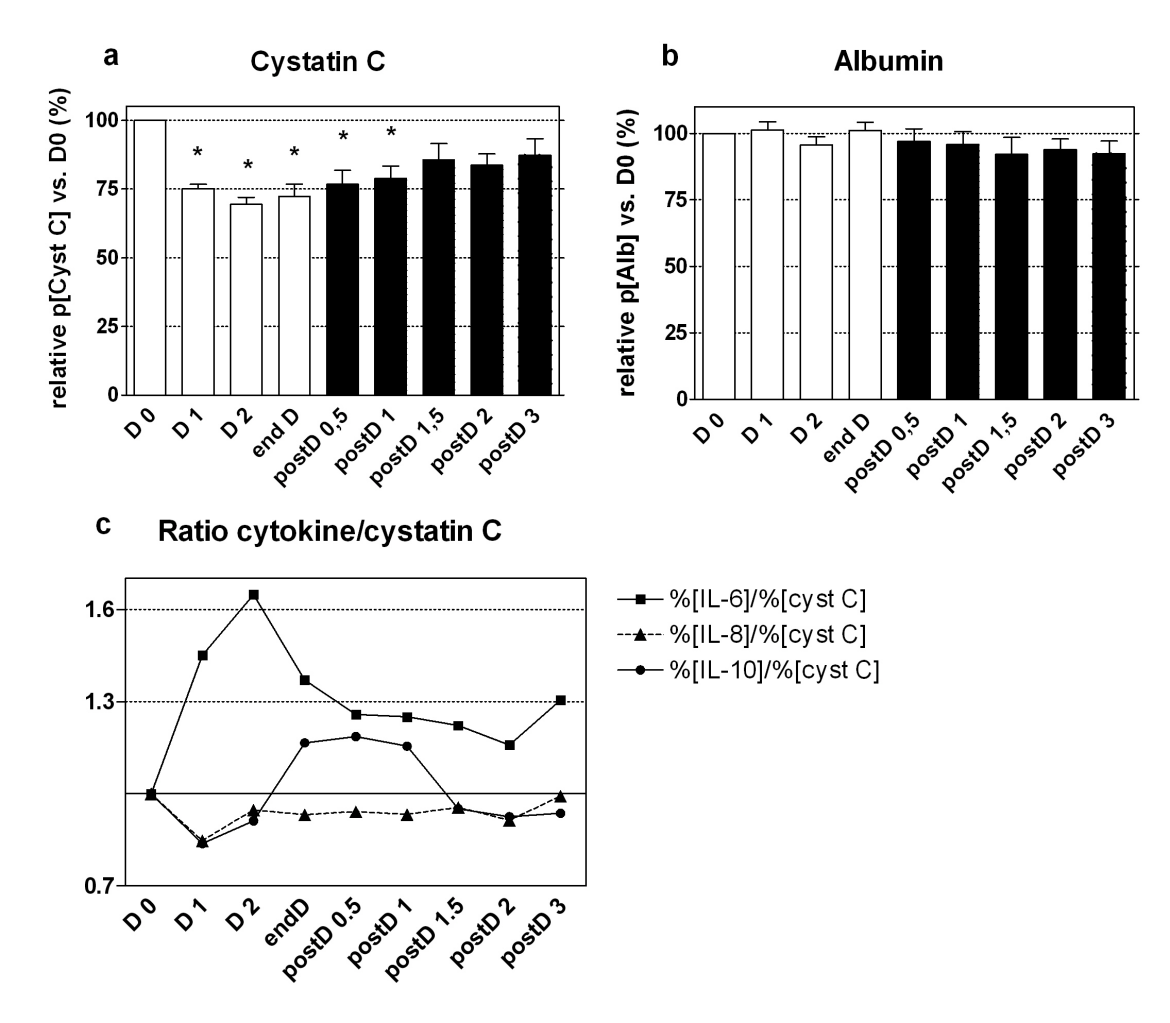
**Protein variations during and after hemodialysis**. Cystatin C **(a) **and albumin **(b) **variations during (clear) and after (dark) hemodialysis. Results are expressed in percentage of value at D0 as mean ± SEM. **(c) **Ratio of percentage of value at D0 of IL-6, IL-8, and IL-10 versus cystatin C. IL-6 (about 26 kDa), IL-8 (about 8 kDa), cystatin C (about 13 kDa), IL-10 (about 19 kDa), and albumin (about 68.5 kDa). *P < 0.05 versus D0; Friedman test.

At D0, the plasma concentrations of IL-6, IL-8, and IL-10 were 840 ± 540, 666 ± 586, and 178 ± 173 pg/ml. Interleukins, cystatin C, and albuminemia plasma concentrations are expressed as variations from the baseline value (D0) (Figures 2 and 3).

During HD, p[IL-6] did not vary significantly, whereas p[IL-8] decreased with hemodialysis, followed by a progressive increase from endD to postD3. At D1, endD, and postD3, p[IL-8] was 68.2 ± 21.2 (vs. D0, *P *< 0.05), 71.1 ± 21.7, and 96.3 ± 35.3%, respectively.

After a maximal decrease at D1, p[IL-10] increased between D2 and postD1.5. The p[IL-10] at D1, endD, and postD3 was 63.7 ± 26, 83.3 ± 68.7 (vs. D0, *P *< 0.05), and 83.3 ± 55.6%, respectively (see Figure 2).

p[Cystatin C] was significantly reduced from D1 to postD1 with a maximal reduction of 30 ± 6.7% at D2 (vs. D0, *P *< 0.05), whereas no modification of albuminemia occurred within the study period (*P *= ns; see Figure 3a, b).

## Discussion

Inside and outside the hospital, severe sepsis and septic shock remain challenging for practitioners, given their high mortality, often related to multiple organ failure, including renal loss of function [1]. High plasma IL-6, IL-8, and IL-10 levels have recently been associated with increased mortality in human sepsis-related ARF [31]. According to the concept of "peak concentration," intensivists try to decrease all circulating mediators at high plasma concentrations, including pro- and antiinflammatory molecules [32]. Hemofiltration (CRRT) is supposed to be the best way to remove cytokines compared with hemodialysis (IRRT), which is based mainly on diffusion. Thus, the efficiency of plasma cytokines removal has mostly been assessed during CRRT. To our knowledge, only one trial has described the kinetics of plasma cytokines during IRRT for septic shock-related AKI [15], and data concerning plasma cytokine levels after hemodialysis are lacking.

In our study, we observed that for IRRT, membranes that leak proteins only partially and transiently decreased plasma IL-8 and IL-10, although not IL-6 levels. In our ten patients, plasma IL-8 and IL-10 decreased rapidly (before D2) and significantly after the beginning of IRRT (see Figure 2). Interestingly, plasma IL-10 levels started to increase before the end of hemodialysis. We did not analyze the effluent and the membrane, but we suggest that dramatic coating of the membrane with IL-10 (and other middle-mass proteins) had occurred. This coating could have led to the early decrease in the adsorptive properties of the PMMA membrane. As IL-10 is larger than IL-8 (19 vs. 8 kDa, respectively), its removal from the PMMA membrane is probably based mainly on adsorption. In a recent study, Nakada et al. have shown prolonged cytokine elimination during CRRT when using a PMMA-based hemodialyzer, but the extent of adsorption and convection clearance were not clarified [26]. In another way, other molecules, which might have participated in the generation of IL-8 and IL-10, could have been removed. Their removal, rather than having a direct effect on cytokines, might have been responsible for our findings. Finally, a high level of IL-6 or IL-10 generation that was sufficient to exceed removal might have been partially responsible for the lack of cytokine elimination. However, two findings argue against this hypothesis: plasma IL-6 did not increase after IRRT, and the ratio between plasma cytokines (especially IL-8) and cystatin C levels suggests a weight-dependent removal. Cystatin C production is constant, and its variations are almost independent of sepsis [18]. Altogether, these data suggest that clearance of IL-6 and IL-10 was absent and transient, respectively.

As mentioned earlier, the kinetics of plasmatic cytokine levels after IRRT in patients with septic shock-related ARF have not been previously reported. Given the large amount of cytokines produced during sepsis, we hypothesize that cytokines may also be affected by the rebound phenomenon of small molecules (that is, urea and potassium), which occurs within the first hour after intermittent dialysis for chronic renal failure [16]. In brief, the rebound phenomenon is related to the shift of soluble molecules from tissues to the intravascular compartment through a concentration gradient until a new equilibrium occurred. Cytokines are heavier and less diffusive molecules than urea or potassium; thus, this rebound could reflect a cytokine concentrations gradient between tissue and vascular compartments that appeared during hemodialysis. After HD, a progressive release of cytokines from dialysis-induced hypoperfused tissue to the intravascular compartment may occur until a new equilibrium is reached. In these ten patients, we observed an upward trend (but not significant) of p[IL-8] and p[IL-10] (+15.2 and +10.45%, respectively) within the first 90 minutes after hemodialysis. Of note, the interindividual plasmatic cytokines variability may have hampered these data from reaching significance, and thus from revealing a statistically significant cytokine rebound. Nevertheless, we observed that all the cytokines we tested for returned to baseline values after postD3, highlighting for the first time the transient effect of hemodialysis on plasma cytokine concentration.

In our study, we showed that plasma cystatin C was significantly decreased during hemodialysis when using a PMMA membrane, with a maximal reduction of 30%, almost equal to IL-10. This decrease in cystatin C is mostly explained by its molecular mass of about 13 kDa and the *in vivo *filtration cutoff of the BK-1,6 F membrane, estimated at 20 kDa (data provided by the manufacturer). This finding, which should be confirmed by further studies, highlights the inability of cystatin C to assess rGFR in patients with ARF treated with a PMMA hemodialyzer.

Mortality was high (60%) but correlated with disease severity. We used previously described IRRT modalities adapted to hypotensive patients [25]. Herein, although our study was not designed to analyze clinic features, we did not identify any worsening of hemodynamic parameters (MAP, HR, NE infusion, SvO_2_) during HD (see Figure 1a, b). Moreover, amounts of NE infused at postD3 were significantly decreased versus D0 (*P *< 0.01, Figure 1b), without any significant fluid-loading challenge. Cytokine removal is thought to be the major component of the beneficial effect of RRT in sepsis, but, in our study, improvement of hemodynamic status was not correlated with cytokine reduction [33-35]. This last observation is in agreement with a study conducted by Klouch *et al*. [36], in which hemodynamic improvement during CRRT was not correlated with TNF-α and IL-6 removal.

## Conclusions

Our results, which should be confirmed in larger cohort, strongly suggest that intermittent hemodialysis with PMMA-based membranes decreases plasma IL-8 and IL-10 concentrations (in contrast to IL-6). Moreover, we showed for the first time that only 3 hours after IRRT, cytokine concentrations revert to baseline levels after an initial rebound. These results highlight that IRRT is not associated with prolonged plasma cytokine reductions during septic shock. Finally, as the plasma cystatin C level is reduced during IRRT, it is probably not a valid residual GFR marker during septic ARF requiring IRRT.

## Key messages

• Cystatin C is not an accurate residual glomerular filtration rate marker, as the cystatin C plasma value is reduced during hemodialysis with a PMMA-based membrane.

• Hemodialysis with a PMMA-based membrane decreases IL-8 and IL-10 (but not IL-6) plasma levels in septic shock patients.

• The decrease in IL-8 and IL-10 plasma levels is transient, as 3 hours after hemodialysis, plasma IL-8 and IL-10 levels have reverted to baseline values.

## Abbreviations

AKI: acute kidney injury; D: dialysis; ELISA: enzyme-linked immunosorbent assay; endD: end of dialysis; HD: hemodialysis; HR: heart rate; ICU: intensive care unit; IL: interleukin; IRRT/CRRT: intermittent/continuous renal-replacement therapy; MAP: mean arterial pressure; NE: norepinephrine; p: plasma; PETIA: particle-enhanced turbidimetric immunoassay; PiCCo: pulse-indexed continuous cardiac output; PMMA: polymethymethacrylate; postD: after dialysis; rGFR: residual glomerular filtration rate; SAPS 2: Simplified Acute Physiology Score; SIRS: systemic inflammatory response syndrome; SOFA: Sequential Organ Failure score; SvO_2_: mixed venous oxygen saturation.

## Competing interests

The authors declare that they have no competing interests.

## Authors' contributions

NM, LL, and MBN designed the study. NM and LL coordinated the study. NM was responsible for patient recruitment, blood sample collection, and data acquisition. NM and LL were involved in the interpretation of the data and manuscript drafting. AJ performed cystatin C dosing. OC, NK, VM, JMC, and LR reviewed the manuscript. All authors read and approved the final manuscript.
